# Application of Combined Two-Dimensional and Three-Dimensional Transvaginal Contrast Enhanced Ultrasound in the Diagnosis of Endometrial Carcinoma

**DOI:** 10.1155/2015/292743

**Published:** 2015-05-18

**Authors:** Hui-li Zhou, Hong Xiang, Li Duan, Gulinaer Shahai, Hui Liu, Xiang-hong Li, Rui-xue Mou

**Affiliations:** Department of Ultrasound of Obstetrics and Gynecology, The First Affiliated Hospital, Xinjiang Medical University, Urumqi 830054, China

## Abstract

*Objective*. The goal of this study was to explore the clinical value of combining two-dimensional (2D) and three-dimensional (3D) transvaginal contrast-enhanced ultrasounds (CEUS) in diagnosis of endometrial carcinoma (EC). *Methods*. In this prospective diagnostic study, transvaginal 2D and 3D CEUS were performed on 68 patients with suspected EC, and the results of the obtained 2D-CEUS and 3D-CEUS images were compared with the gold standard for statistical analysis. *Results*. 2D-CEUS benign endometrial lesions showed the normal uterine perfusion phase while EC cases showed early arrival and early washout of the contrast agent and nonuniform enhancement. The 3D-CEUS images differed in central blood vessel manifestation, blood vessel shape, and vascular pattern between benign and malignant endometrial lesions (*P* < 0.05). Sensitivity, specificity, positive predictive value, negative predictive value, and accuracy of transvaginal 2D-CEUS and 2D-CEUS combined with 3D-CEUS for diagnosis of benign and malignant endometrial lesions were 76.9%, 73.8%, 64.5%, 83.8%, and 75.0% and 84.6%, 83.3%, 75.9%, 89.7%, and 83.8%, respectively. *Conclusion*. 3D-CEUS is a useful supplement to 2D-CEUS and can clearly reveal the angioarchitecture spatial relationships between vessels and depth of myometrial invasion in EC. The combined use of 2D and 3D-CEUS can offer direct, accurate, and comprehensive diagnosis of early EC.

## 1. Introduction

Endometrial carcinoma (EC) is the second most predominant cancer of the female reproductive tract, typically occurring in perimenopausal women around 50 years of age. Its incidence rate shows a rising tendency worldwide, yet the 5-year survival rate has declined [1]. EC is one of the most serious problems threatening women's health worldwide. Ultrasonography, hysteroscopy, curettage, and other ways have been widely used in differential diagnoses of endometrial lesions. However, the rates of misdiagnosis and missed diagnosis are as high as 10%–35% [2]. The ways of the accuracy need to be further improved. The depth of myometrial invasion is an important factor affecting the 5-year survival and recurrence of endometrial cancer. Curettage scraping is the most commonly used and most valuable diagnostic methods. Curettage has the benefit of early diagnosis in endometrial carcinoma but demonstrates a degree of difficulty when evaluating the myometrial invasion. Hysteroscopy is considered the gold standard in the diagnosis of intrauterine lesions [2], but it is an invasive examination method and cannot evaluate the degree of myometrial invasion. MRI clearly shows the uterus and pelvic lymph nodes of each layer structure; this is the most reliable method of identifying cervical involvement, but it is unpredictable in estimating the depth of myometrial invasion 79.2%~91.4% [3, 4]. PET/CT in pelvic lymph node metastasis in endometrial cancer shows a huge advantage, especially for the metastasis lymph nodes >5 mm in diameter [5]. However, the examination method is expensive and has an extensive test time; furthermore, there is the risk of allergy to the contrast agent. Beyond this, the method is widely limiting for patients with specific requirements. Saline infusion sonohysterography improves the rate of diagnosis of endometrial carcinomas, but that process may disseminate malignant cells into the peritoneal cavity through the fallopian tubes [6]. Correct preoperative identification of the nature of endometrial lesions and proper assessment of the depth of myometrial invasion EC are issues of common interest for both clinicians and ultrasound physicians. Though the transvaginal ultrasound examination has become the most common method, it is performed in real time, involves no radiation, and is inexpensive and noninvasive. However, it can only provide information relating to tumor vessels and the blood supply and distribution within the macroscopic evaluation of lesions; probing and displaying some of the microcirculation and small blood vessels result in an undesirable effect [7]. With the rapid development of contrast-enhanced ultrasound imaging and related technologies, it is now possible to diagnose disease from the level of organization in a microcirculation perfusion study, which has greatly improved the accuracy of an ultrasonic diagnosis. We can better detect the blood flow in small, deep vessels, and this in turn improves the ability to differentiate between areas of normal and abnormal perfusion [8]. This study was to evaluate the utility of contrast-enhanced Sonography. Contrast agents is SonoVue, it is a kind of blood pool and it can reflect the blood flow perfusion of sensitive information and improve the accuracy of disease diagnosis [9]. The goal was to determine the clinical value of preoperative transvaginal CEUS in identifying the nature of endometrial lesions and assessing the depth of myometrial invasion to provide a basis for preoperative selection of proper treatment options in clinical practice.

## 2. Materials and Methods

### 2.1. Subjects

Sixty-eight patients with clinically suspected EC were subjected to CEUS examinations in the Department of Ultrasound in Obstetrics and Gynecology, First Affiliated Hospital of Xinjiang Medical University, from January 2013 to February 2014. The patients were 23–78 years of age, with a mean age of 50.72 ± 12.90 years. All patients signed an informed consent, which had been approved by the ethics committee before CEUS.

### 2.2. Instruments and Methods

MyLab90 color Doppler (Esaote SpA, Genoa, Italy) ultrasonography with supporting CnTI imaging software and 3D reconstruction software was used. The probe model was EC123, with a frequency of 3.0–9.0 MHz and a mechanical index (MI) of 0.08. SonoVue contrast agent was used (Bracco SpA, Milan, Italy). For use of a contrast agent, 59 mg SonoVue was added to 5 mL saline and mixed well. A bolus of 2.4 mL was injected into the median cubital vein and was immediately followed by injections of 5–10 mL saline. After emptying the bladder, each patient assumed a lithotomy position and a conventional 2D transvaginal ultrasonography of the uterus, ovaries, and pelvis was performed. The endometrial thickness and uterine morphology, the presence or absence of abnormal intrauterine lumps or fluid, tumor invasions in the myometrial, cervical or parauterine tissues, and the endometrium as well as blood flow in the endometrial lesion were examined. After the sagittal plane of the uterus and endometrial morphology were clearly revealed in the 2D mode, CEUS mode was begun with the suspected myometrial invasion or the deepest invasion serving as the section of interest. After 2.4 mL of contrast agent had been injected into the cubital vein, the lesion was observed continuously for over 3 min. After 10 min, scanning of the lesion was performed in dynamic 3D mode, and the images were stored. Two experienced physicians analyzed images obtained from 68 patients, in a double-blind fashion, independently, using 3D software. The perfusion characteristics of the contrast agent, intensity of enhancement, enhancement start time, peak time, contrast agent washout time, and lesion border, as well as endometrial thickness, lesion range, thickness of normal uterine myometrium, and thickness of myometrial invasion, were determined. For all 68 patients, surgery was performed within one week after the CEUS examination, and the ultrasound results were compared with results of surgical pathology.

### 2.3. Diagnostic Criteria in the Experiment

*(1) CEUS Diagnostic Criteria for EC*. The endometrial perfusion shows “early in, early out” regarding the contrast agent, that is, early arrival, early peak, and early washout of the contrast agent; regarding intensity and uniformity of enhancement, EC was manifested using nonuniform high enhancement [10, 11].

*(2) Assessment of Tumor Myometrial Invasion Depth*. In 3D-CEU, the difference between the thickness of normal myometrium (i.e., the distance between endometrial-myometrial interface and serosa) and the distance from the deepest lesion invasion to serosa is defined as the depth of lesion invasion. According to the ratio of this depth to the thickness of the normal myometrium, invasion depth can be divided into the following two categories: <1/2 and ≥1/2 [12].

### 2.4. Analysis of Blood Flow Signals

*(1) Vascular Pattern [13]*

*Type I*. There is no blood flow signal surrounding or within the tumor.

*Type II*. There are blood flow signals in the shape of dots or lines surrounding the tumor and no blood flow signal within the tumor.

*Type III*. In addition to surrounding blood vessels, there are sparse blood vessels within the tumor, with simple branching and a relatively straight course.

*Type IV*. There is relatively rich vascular tree or vascular network within the tumor, with complex branching, tortuous and irregular shapes, and blood vessels surrounding the tumor.

*(2) Analysis of Time-Intensity Curve*. The time intensity of the tumor section was measured prior to the angiography and continuously after injection of the contrast agent until the intensity recovered to the level before the angiography. There were three crucial temporal parameters of the CEUS blood flow images, which included the following: enhancement start time, peak time, and transit time.

### 2.5. Statistical Analysis

SPSS17.0 statistics software was used for data analysis. Quantitative data are expressed as mean ± standard deviation. For qualitative data, *χ* ^2^ test and rank sum test were performed. *P* < 0.05 was considered statistically significant. The sensitivity (Sen), specificity (Sep), positive predictive value (PV+), negative predictive value (PV−), positive likelihood ratio (LR+), negative likelihood ratio (LR−), and accuracy rate of 2D-CEUS and 2D-CEUS combined with 3D-CEUS in diagnosing the nature of endometrial lesion and assessing the myometrial invasion depth in EC (<1/2, ≥1/2) were determined.

## 3. Results

### 3.1. Basic Clinical Information

Among the 68 patients diagnosed with endometrial lesions using a conventional transvaginal ultrasound, there were 32 cases of endometrial thickening, with endometrial thickness of 0.5–3.0 cm and a mean thickness of 2.05 ± 0.87 cm. There were 23 cases of intrauterine lesions with abnormal echoes and unclear boundaries between the lesion and the surrounding myometrium, 13 cases of endometrial thickness <0.5 cm and uneven endometrial echoes, and 15 cases of endometrial lesion with concomitant uterine fluid.

### 3.2. Pathology Results of the 68 Cases of Suspected EC

There were 42 cases of benign endometrial lesions with 19 cases of endometrial polyps, 10 cases of endometrial hyperplasia, 3 cases of submucosal uterine fibroids, 4 cases of uterine fluid and blood accumulation, and 6 cases of cystic endometrial atrophy. There were 26 cases of EC with 18 cases of tumor myometrial invasion to depths of <1/2 and 8 cases to a depth of ≥1/2.

### 3.3. CEUS Results

**Figure 1:**
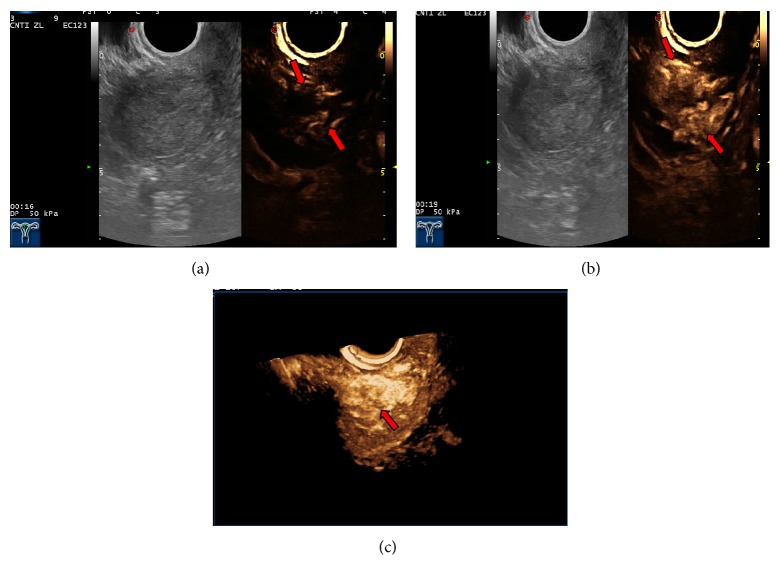
Endometrial carcinoma with superficial myometrial invasion Stage IA. (a) Image at the 16th second after administration showed blood flow signals in the shape of lines (arrows). (b) Image at the 19th second showed the maximal concentration of contrast agent in the tumour (arrows). (c) Image of 3D-CEUS shows tortuous, irregular blood vessels twisted into a group (arrow).

(1) CEUS manifestations of benign endometrial lesion: the characteristics of the contrast agent perfusion in the 42 cases of benign endometrial lesion are shown in Table 1. 3D-CEUS clearly revealed abnormal intrauterine space-occupying lesions; particularly in the coronal section it showed the continuity of endometrial stripe, the morphology of endometrial edge, and the intact basal layer of endometrium and directly revealed the spatial relationship between the lesion and the myometrium. For these benign endometrial lesions, 3D-CEU showed straight blood vessels of regular shapes near the lesion and sparse blood vessel distribution within the lesion, and the vascular patterns were mostly types II and III (see Table 2).

(2) CEUS manifestations of EC: the lesions in 25 cases of EC showed medium-high enhancement. A small amount of thickened, irregular nourishing blood vessels from the myometrium to the uterine cavity was found, and the involved myometrium mainly exhibited uneven enhancement. During the washout period, washout of the contrast agent was faster than in normal myometrium, and the boundary between the lesion and the myometrium was clear. The depth of myometrial invasion was determined in the washout period. In 14 cases, the involved myometrium had a thickness of <1/2 of the myometrial thickness, and in eight cases the involved myometrium had a thickness of ≥1/2 of the myometrial thickness. In three cases, low enhancement and a normal myometrial perfusion phases were found. Imaging and reconstruction of the spatial relationships between tissues supplemented the diagnostic ability of 2D-CEUS findings. In one case, EC at a uterine horn overlooked on 2D-CEUS was identified with 3D-CEUS. In two cases, the lesion was found to be larger than previously detected on 2D-CEUS. 3D-CEUS corrected one case that was misdiagnosed as stage IA EC with 2D-CEUS imaging; the correct diagnosis using 3D ultrasound imaging and pathology was endometrial polyps with uterine adenomyosis. 3D-CEU revealed tortuous, irregular blood vessels, twisted into groups (Figure 1), which were predominantly characteristic patterns of types III and IV (Table 2).

The benign and malignant endometrial lesions differed significantly in the perfusion phase of the contrast agent (*P* < 0.05) but not in intensity of enhancement or uniformity of enhancement (*P* > 0.05) (see Table 1).

The 3D-CEUS images of benign and malignant endometrial lesions differed significantly in display of central blood vessels, blood vessel shapes, and vascular patterns (*P* < 0.05) (see Table 2).

**Figure 2:**
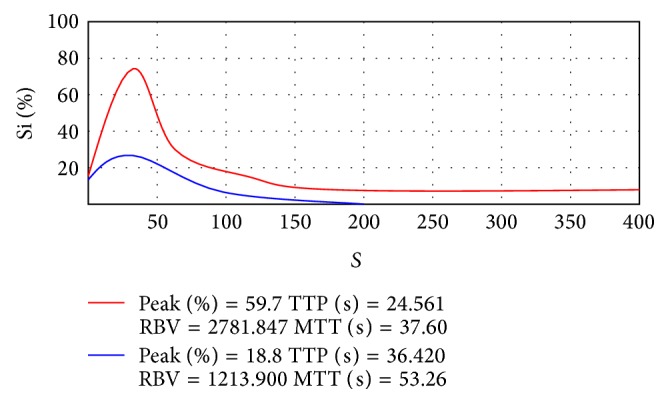
The time-intensity curve for endometrial lesion. Red curve: the time-intensity curve for endometrial carcinoma, rapid rise, and rapid decline, with a sharp peak. Blue curve: the time-intensity curve for benign endometrial lesion, rapid rise, and slow decline, with a blunt peak.

(3) Time-intensity curve of CEUS: in the malignant endometrial lesion group, the time-intensity curves showed an overall shape of “rapid rise and rapid decline” with a peak sharp (Figure 2). In the benign endometrial lesion group, the time-intensity curves showed an overall shape of “rapid rise and slow decline” with a round and blunt peak (Figure 2). Regarding the angiographic parameters, the two groups differed significantly in the enhancement start time (*P* < 0.05) but not in the peak time or transit time (*P* > 0.05) (see Table 3).

(4) The diagnoses performance of endometrial lesions using transvaginal 2D-CEUS and combined 2D- and 3D-CEUS are illustrated in Table 4.

### 3.4. The Clinical Value of Combined 2D and 3D Transvaginal CEUS in the Assessment of Myometrial Invasion Depth in EC

**Figure 3:**
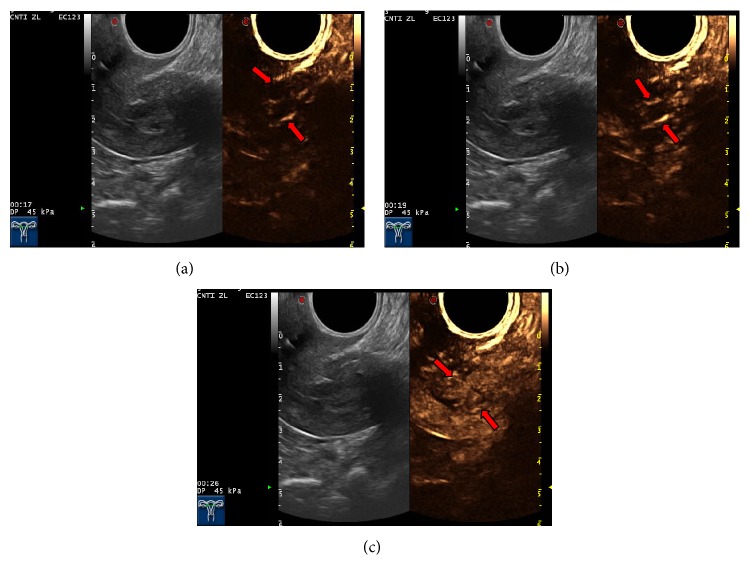
Endometrial hyperplasia and myoma of uterus misdiagnosed as stage IB endometrial cancer. (a), (b), and (c) represent the first 16 seconds, 19 seconds, and 26 seconds, respectively, showing two thick blood vessels (b) extending from fundal myometrium to uterine cavity and the maximal concentration of contrast agent (c) in the tumour.

**Figure 4:**
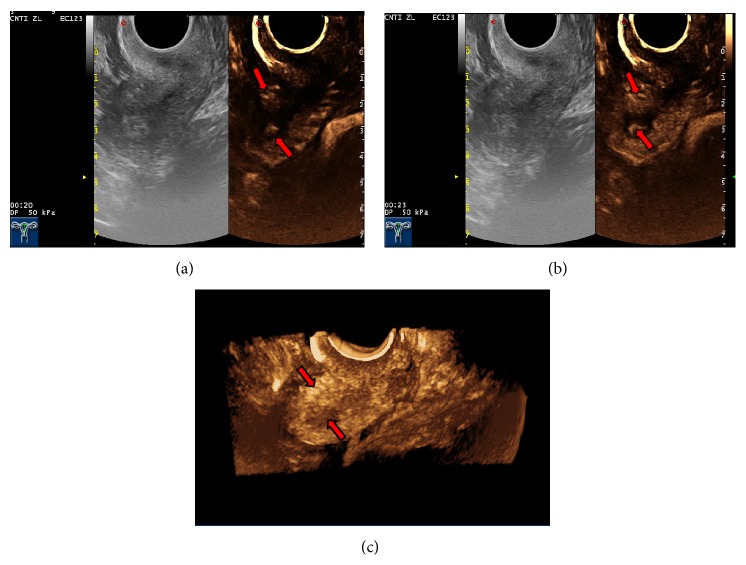
Adenomyosis with concomitant endometrial polyp was mistakenly diagnosed as EC. Image at 20 and 23 seconds and 3D-CEUS showed that the adenomyosis was manifested by a locally enhanced echogenic area, and its boundary with the uterine cavity was unclear; it was thus misdiagnosed as deep myometrial invasion in endometrial carcinoma.

(1) Characterization of endometrial lesions using CEUS: all the 68 patients with suspected EC underwent preoperative 2D-CEUS and 3D-CEUS examinations. With 2D-CEUS, 29 cases were diagnosed with EC; of these, 24 cases were correctly diagnosed. Five cases with benign endometrial lesions were mistakenly diagnosed as EC (three cases of endometrial polyps, one case of submucosal fibroid, and one case of endometrial hyperplasia). In a mistakenly diagnosed case (endometrial hyperplasia with concomitant uterine fibroid), 2D-CEUS revealed two thick blood vessels extending into the uterine cavity (Figure 3). Two cases of EC were mistakenly diagnosed as benign uterine lesions (one case of stage IA EC was diagnosed as endometrial hyperplasia, and another case of stage IA EC was diagnosed as an endometrial polyp). With 3D-CEUS, 30 cases were diagnosed with EC, and, of these, 25 cases were correctly diagnosed. In one case, adenomyosis with concomitant endometrial polyp was mistakenly diagnosed as EC (Figure 4), and in two cases, EC was mistakenly diagnosed as an endometrial polyp.

(2) Assessment of myometrial invasion depth in malignant endometrial lesions using CEUS: diagnostic results on the depth of myometrial invasion in EC using 2D-CEUS and combined 2D- and 3D-CEUS are shown in Table 5.

(3) The quality of the diagnosis on the depth of myometrial invasion in EC using 2D-CEUS and combined 2D- and 3D-CEUS is illustrated in Table 6.

## 4. Discussion

EC and benign endometrial lesions are both common gynecological diseases. The clinical manifestations of the two are very similar, yet the clinical treatment methods as well as prognoses are completely different. Therefore, a correct differential diagnosis between benign and malignant endometrial lesions and accurate assessment of myometrial invasion in EC are crucial for clinical practice. It is difficult to determine the nature of endometrial lesions using conventional ultrasound [14]. CEUS can clearly reveal tiny blood vessels in the lesions and tumor perfusion and allow the continuous observation of the contrast agent in the lesions from arrival to washout. Further image processing can isolate and cancel out base level signals from normal tissues and display the contour of the lesion [15, 16]. Luo et al. [17] reported that the accuracy of diagnosing endometrial thickening using CEUS was significantly improved compared to conventional ultrasound. Here, we found that the accuracy of characterization of endometrial lesions using combined 2D-CEUS and 3D-CEUS (83.8%) was higher than using 2D-CEUS alone (75.0%). Malignant and benign endometrial lesions were found to differ during the perfusion phase of the contrast agent, which is consistent with the findings reported by Chen et al. [18]. It has been shown that [19], among all diagnosed EC patients, 75%–80% cases are stage I EC. Zamani et al. [20] reported that the surgical staging criteria revision by FIGO in 2009 improved the accuracy of preoperative staging of early EC patients. Hence, the focus of diagnosis has changed from determining whether there is myometrial invasion to assessing the myometrial invasion depth. The lymph node metastasis rate of stage IA EC is less than 5%, and thus typically no retroperitoneal lymph node dissection is performed. Yet, for stage IB EC patients, resection of uterus and bilateral attachments as well as retroperitoneal lymph node dissection is performed [21]. Hence, a preoperative diagnosis must not only differentiate between benign and malignant endometrial lesions, but also accurately determine myometrial invasion depth in EC. A study by Liu et al. [11] reported that compared to conventional ultrasound CEUS showed a higher accuracy rate in assessing the depth of myometrial invasion (67.1%). This is a blow to our findings (the accuracy rate of 2D-CEUS diagnosis was 75.9%; that of combined 2D-CEUS and 3D-CEUS was 82.8%). However, Pei et al. [22] suggested that CEUS was not significantly more accurate when assessing the myometrial invasion depth in the diagnosis of stage I EC.

**Figure 5:**
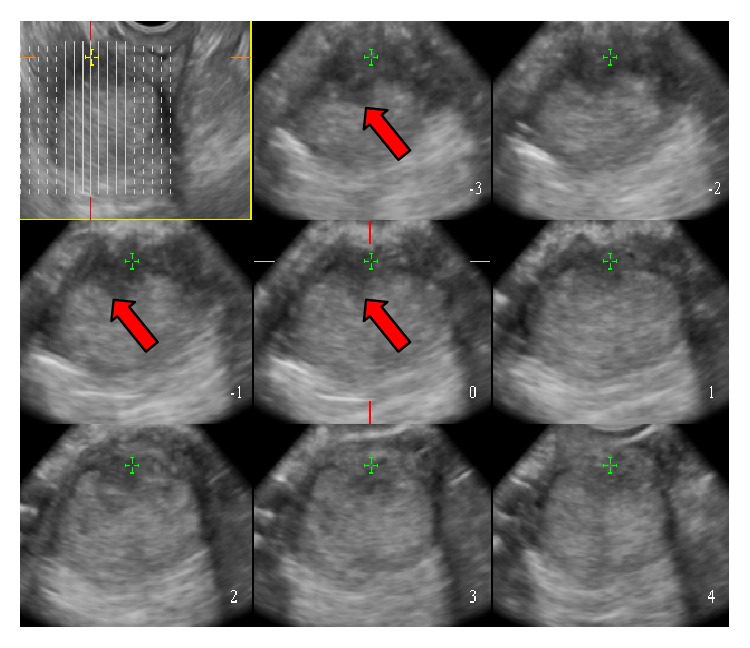
3D-CEUS reveals the spatial relationship between the intrauterine lesion and the surrounding myometrium clearly (arrows).

**Figure 6:**
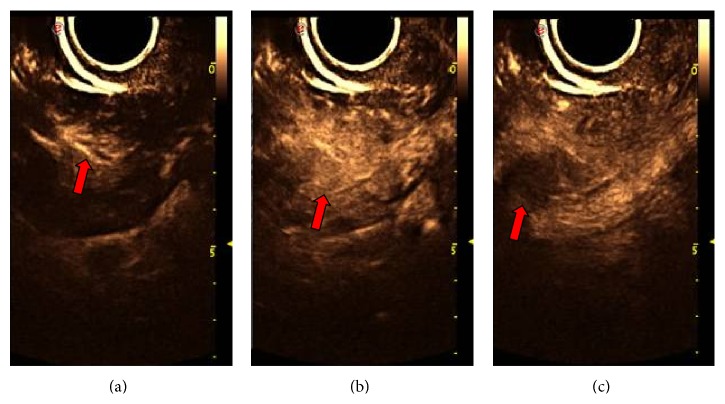
Endometrial carcinoma with superficial myometrial invasion Stage IA. Image at 20, 22, and 40 seconds showed that during the arterial phase of CEUS (a) the nourishing artery of the lesion is enhanced at first, simultaneously followed by the lesion and uninvolved myometrium (b). During the venous phase, flow-out of the agent in the lesion is slightly faster than that in the uninvolved myometrium, resulting in a hypoechoic appearance (c), which can be used to identify the depth and scope of endometrial carcinoma.

**Figure 7:**
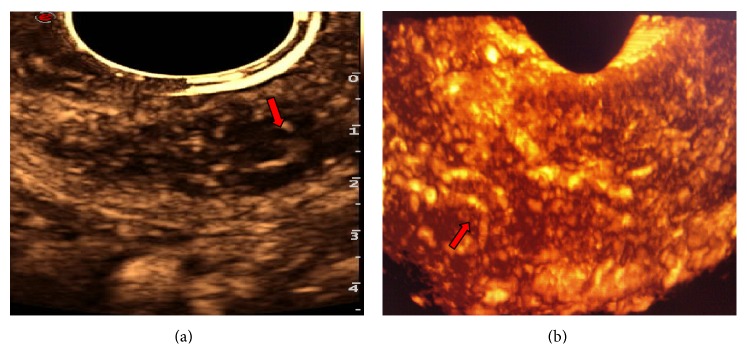
Endometrial carcinoma with superficial myometrial invasion. (a) Blood flow signals in 2D-CEUS are in the shape of dots and line segments (arrow). (b) Blood flow signals in 3D-CEUS reveal clear blood vessels with twisted shapes (arrows).

**Figure 8:**
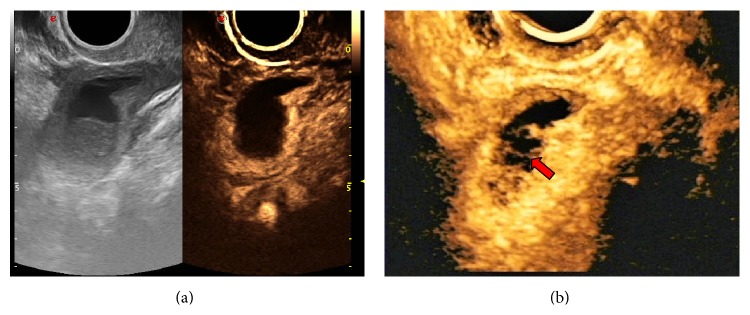
Endometrial carcinoma with superficial myometrial invasion Stage IA. 3D-CEUS reveals tiny nourishing blood vessels (arrow) that 2D-CEUS fails to reveal.

Transvaginal 2D-CEUS is the preferred method for examination of endometrial lesions. Yet, endometrial lesions are typically irregularly shaped, and transvaginal 3D-CEUS images are direct, clear, and of course three-dimensional [23] and therefore capable of displaying the size and morphology of the lesion from all angles and its spatial relationship with the endometrial wall (Figure 5). Thus, 3D-CEUS imaging is superior to 2D-CEUS in revealing endometrial lesions and can provide a reliable basis for diagnosis [24]. In particular, 3D-transvaginal sonography (3D-TVS) has special advantages in assessing myometrial invasion in EC. However, the endometrial lesion is smaller than the myometrium, and the two are in close proximity to each other. Conventional sonography cannot clearly depict the precise location of the lesion, making it difficult to accurately evaluate the depth of myometrial invasion. The developments in CEUS and related technologies have improved the capability of ultrasound in lesion detection and qualitative diagnosis. During the enhancement period, the base of the lesion can be determined with relative ease, and, during the washout period, the boundary between the lesion and normal myometrium is clear, which helps determine the presence of myometrial invasion and its depth (Figure 6). Pei et al. [22] showed that CEUS could accurately reflect blood perfusion in EC cases. Aboul-Fotouh et al. [25] reported that color Doppler energy images could be used for differential diagnosis between benign and malignant endometrial lesions and were especially accurate in the diagnosis of EC, which is characterized by a type C vascular pattern. From this it can be inferred that vascular patterns of EC revealed by CEUS are predominantly of types III and IV. 2D-CEUS can only reveal the blood supply distribution in one section of the lesion, and small tortuous vessels are displayed as dots of varying sizes or strips of varying lengths (Figure 7(a)). Hence, 2D-CEUS cannot fully reflect the blood supply within the entire lesion. 3D-CEUS can overcome this problem and obtain relatively complete three-dimensional microcirculation images of the lesion, thereby making the structure of the vascular tree clear for observation (Figure 7(b)). Furthermore, 3D-CEUS shows the three-dimensional dynamic perfusion of the contrast agent in the endometrial lesion and improves the resolution on the details of the microcirculation within the lesion (resolution on the lesions, the blood vessels, and the vascular spatial relations) (Figure 8). Transvaginal 3D-CEUS examination is a new examination method combining 3D-TVS and CEUS. It clearly displays the entire intrauterine coronal section and allows accurate assessment of myometrial invasion depth. Hence, its clinical application has a promising prospect [26].

In summary, the application of 3D-CEUS in the diagnosis of endometrial lesions is still in its infancy. However, it has already shown its ability to correct differential diagnosis between benign and malignant endometrial lesions and accurately assess myometrial invasion in EC. Meanwhile, 3D-CEUS is the combination of contrast-enhanced ultrasonography and three-dimensional imaging technology. Image acquisition, reconstruction, and processing may introduce artifacts causing image distortion, leading to misinterpretation [27]. Great care should be taken to avoid such diagnostic errors. Additional studies are warranted to further evaluate the efficacy of 3D-CEUS in improving early diagnosis of endometrial carcinoma.

## 5. Conclusion

Transvaginal 3D-CEUS is a supplement to 2D-CEUS. Compared to 2D-CEUS, 3D-CEUS is more accurate in revealing endometrial abnormalities, diagnosing endometrial lesions, and assessing the depth of myometrial invasion and is therefore of important value for early diagnosis of EC. In EC diagnosis, transvaginal 2D-CEUS can be used as a basic examination for real-time dynamic observation of tumor perfusion. Next, 3D imaging can be applied to understand the three-dimensional structure of the lesion and the overall vascular structure in the lesion to provide a direct, accurate, and comprehensive basis for the final diagnosis.

## Figures and Tables

**Table 1:** Features of CEUS images of benign and malignant endometrial lesions (number of cases).

Nature of lesion	Number of cases	Perfusion phase of contrast agent			Intensity of enhancement			Uniformity of enhancement	
		Early in early out	Early in late out	Normal in normal out	High	Medium	Low	Even	Uneven
Benign	42	19	3	20	17	14	6	29	15
Malignant	26	17	4	5	20	5	3	11	15
Statistics value		−2.009			−1.880			3.717	
*P* value		0.045			0.060			0.054	

Note: *χ* ^2^ test and rank sum test.

**Table 2:** 3D-CEUS images of benign and malignant endometrial lesions (number of cases).

Nature of lesion	Number of cases	Central blood vessel		Blood vessel shape		Vascular type			
		Revealed	Not revealed	Straight	Irregular	I	II	III	IV
Benign	42	10	32	39	3	10	10	17	5
Malignant	26	21	5	8	18	1	5	8	12

Statistic value		21.004		29.002		−3.105			
*P* value		0.000		0.000		0.002			

Note: *χ* ^2^ test and rank sum test.

**Table 3:** Parameter analysis of the time-intensity curves for endometrial lesions.

Nature of lesion	Number of cases	Enhancement start time (ms)	Peak time (s)	Transit time (s)
Benign	42	16200.00 (4950.000)	25.46 (13.405)	43.08 (17.490)
Malignant	26	12960.00 (3810.000)	23.83 (9.360)	38.13 (16.325)

Statistics value		−2.581	−1.011	−1.117
*P* value		0.010	0.312	0.907

Note: rank sum test.

**Table 4:** The diagnoses performance of endometrial lesions using transvaginal 2D-CEUS and combined 2D- and 3D-CEUS.

Methods	Sen	Spe	LR+	LR−	PV+	PV−	Accuracy rate
2D	76.9%	73.8%	1.81	0.40	64.5%	83.3%	75.0%
2D + 3D	84.6%	83.3%	5.07	0.18	75.9%	89.7%	83.8%

Sen: sensitivity.

Spe: specificity.

LR+: positive likelihood ratio.

LR−: negative likelihood ratio.

PV+: positive predictive value.

PV−: negative predictive value.

**Table 5:** Diagnosis on the depth of myometrial invasion using combined transvaginal 2D- and 3D-CEUS.

Method	Ultrasound diagnosis	Staging		Total
		<1/2	≥1/2	
2D-CEUS	<1/2	16	4	20
	≥1/2	3	6	9
2D + 3D-CEUS	<1/2	17	2	19
	≥1/2	3	8	11

**Table 6:** Quality of diagnosis on the depth of myometrial invasion using combined transvaginal 2D- and 3D-CEUS.

Methods	Sen	Spe	LR+	LR−	PV+	PV−	Accuracy rate	*K* value
2D	84.2%	60.0%	2.11	0.26	80.0%	66.7%	75.9%	0.453
2D + 3D	85.0%	77.8%	3.83	0.19	89.5%	70.0%	82.8%	0.609

Sen: sensitivity.

Spe: specificity.

LR+: positive likelihood ratio.

LR−: negative likelihood ratio.

PV+: positive predictive value.

PV−: negative predictive value.
